# Comparative secretome analyses of two *Trichoderma reesei *RUT-C30 and CL847 hypersecretory strains

**DOI:** 10.1186/1754-6834-1-18

**Published:** 2008-12-23

**Authors:** Isabelle Herpoël-Gimbert, Antoine Margeot, Alain Dolla, Gwénaël Jan, Daniel Mollé, Sabrina Lignon, Hughes Mathis, Jean-Claude Sigoillot, Frédéric Monot, Marcel Asther

**Affiliations:** 1 INRA, UMR1163, Biotechnologie des Champignons Filamenteux, F-13000 Marseille, France; 2 Universités Aix-Marseille I & II, UMR1163, BCF, F-13000 Marseille, France; 3 IFP, Biotechnology Department, Avenue de Bois-Préau, 92852 Rueil-Malmaison Cedex, France; 4 IMR, FRE3083 – CNRS, Institut de Biologie Structurale et Microbiologie, Chemin Joseph-Aiguier, 13402 Marseille cedex 20, France; 5 INRA, UMR1253, Science et Technologie du Lait et de l'Oeuf, F-35000 Rennes, France; 6 Agrocampus Rennes, UMR1253, STLO, F-35000 Rennes, France; 7 Plate-forme protéomique, Institut de Biologie structurale et Microbiologie, Chemin Joseph-Aiguier, 13402 Marseille cedex 20, France

## Abstract

**Background:**

Due to its capacity to produce large amounts of cellulases, *Trichoderma reesei *is increasingly been researched in various fields of white biotechnology, especially in biofuel production from lignocellulosic biomass. The commercial enzyme mixtures produced at industrial scales are not well characterized, and their proteinaceous components are poorly identified and quantified. The development of proteomic methods has made it possible to comprehensively overview the enzymes involved in lignocellulosic biomass degradation which are secreted under various environmental conditions.

**Results:**

The protein composition of the secretome produced by industrial *T. reesei *(strain CL847) grown on a medium promoting the production of both cellulases and hemicellulases was explored using two-dimensional electrophoresis and MALDI-TOF or LC-MS/MS protein identification. A total of 22 protein species were identified. As expected, most of them are potentially involved in biomass degradation. The 2D map obtained was then used to compare the secretomes produced by CL847 and another efficient cellulolytic *T. reesei *strain, Rut-C30, the reference cellulase-overproducing strain using lactose as carbon source and inducer of cellulases.

**Conclusion:**

This study provides the most complete mapping of the proteins secreted by *T. reesei *to date. We report on the first use of proteomics to compare secretome composition between two cellulase-overproducing strains Rut-C30 and CL847 grown under similar conditions. Comparison of protein patterns in both strains highlighted many unexpected differences between cellulase cocktails. The results demonstrate that 2D electrophoresis is a promising tool for studying cellulase production profiles, whether for industrial characterization of an entire secretome or for a more fundamental study on cellulase expression at genome-wide scale.

## Background

The filamentous cellulolytic fungus *Trichoderma reesei *is known to be an exceptionally efficient producer of cellulases and hemicellulases acting in synergy to degrade lignocellulosic materials. *T. reesei *produces a broad range of cellulases able to hydrolyze the β-1,4 glycosidic bonds present in celluloses and derivatives. The glucose produced provides the fungus with a carbon source readily usable for growth. One of the main applications is the conversion of lignocellulosic biomass to biofuels, such as ethanol [1]. Due to their biotechnological interest, the most abundant hydrolytic enzymes of *T. reesei *have been the subject of extensive structural [2-6] and genetic studies (see reviews [7,8]). These enzymes include two cellobiohydrolases (Cel7A and Cel6A, E.C. 3.2.1.91) that act like exoenzymes, releasing cellobiose from crystalline cellulose as the main product, five endoglucanases (Cel7B, Cel5A, Cel12A, Cel61A and Cel45A, EC 3.2.1.4) that attack cellulose in an endo-acting manner with a strong affinity towards the soluble derivatives of cellulose, and two β-glucosidases (BGLI and BGLII, EC 3.2.1.21) that cleave cellobiose to glucose [9]. The hemicellulolytic system of *T. reesei *is composed of several enzymes, including endo-1,4-β-xylanases (XYNI, 2, 3 and 4, EC 3.2.1.8), mannanase (MANI, EC 3.2.1.78), acetylxylan esterase (AXEI, EC 3.1.1.72), α-galactosidase (BGAI, EC 3.2.1.22) and arabinofuranosidase (ABFI, EC 3.2.1.55) [10-12]. In addition, new biomass degradation-related genes have been identified through cDNA sequencing and DNA microarrays [13,14].

Cellulases are produced industrially using *T. reesei *strains genetically modified by random mutagenesis or by targeted genetic modifications such as introducing strong inducible promoters, increasing the gene copy numbers or removing undesired secreted proteins [15]. Industrial mutant strain CL847 yields a high production (about 40 g L^-1^) of proteins secreted in the extracellular medium [16,17]. Commercial cellulolytic products are generally poorly characterized enzyme mixtures containing cellulases and other unidentified proteins. The sole 2D maps of *T. reesei *secretomes reported in the literature were established from commercial cellulase preparations prior to the availability of genome sequences [18,19], making it difficult to identify new enzymes. The recent ongoing sequencing of the *T. reesei *QM6a strain genome available from the DOE Joint Genome Institute  gives an opportunity to gain a better understanding of the variety of enzymes secreted by this fungus.

We performed a proteomic study of the enzymes secreted from *T. reesei *CL847, which is a strain already used at industrial scale, grown under conditions promoting the production of both cellulases and hemicellulases, using 2D electrophoresis (2DE) gels coupled with MALDI-TOF and LC-MS/MS mass spectrometry. The 2D map obtained was used to compare the secretome composition of CL847 with that of the well-known cellulase overproducer Rut-C30 [20] under cellulolytic enzyme secretion-promoting conditions.

## Methods

### Fungal strain and culture conditions

For storage, *T. reesei *strains CL847 [21] and Rut-C30 (ATCC 56765) cultures were grown on plates of Potato Dextrose Agar (Difco Laboratories, USA) at 30°C. After sporulation, the spores were resuspended in a sterile NaCl (9 g L^-1^)-glycerol 20% solution and stored at -80°C. Frozen spores were used to inoculate a Fernbach flask containing 250 mL of culture medium (glucose 30 g L^-1^; corn steep 2 g L^-1^; (NH_4_)_2_SO_4 _1.4 g L^-1^; KOH 0.8 g L^-1^; H_3_PO_4 _85% 4 mL L^-1^; phthalic acid, dipotassium salt 5 g L^-1^; MgSO_4_.7H_2_O 0.3 g L^-1^; CaCl_2 _0.3 g L^-1^; FeSO_4_.7H_2_O 5.0 mg L^-1^; MnSO_4_.H_2_O 1.6 mg L^-1^; ZnSO_4_.7H_2_O 1.4 mg L^-1^; CoCl_2_.6H_2_O 2.0 mg L^-1^). Cultivation was carried out at 30°C with stirring at 110 rpm. After 72 h, 100 mL of medium broth was used as an inoculum for bioreactor culture. Fermentation of *T. reesei *was carried out in a 4 L bioreactor under culture conditions previously described by Pourquié and Warzywoda, 1993 [17]. The cellulase production was performed in two steps. In the first step, a growth phase, with 2 L starting medium containing 35 g L^-1 ^of lactose as carbon source, 27°C and pH regulated at 4.8 (with 6 M ammonia) was conducted. The air flow was adjusted at 0.5 VVM and initial stirring was set at 500 rpm. This parameter was gradually increased to maintain pO_2 _above 40% oxygen saturation. In the second step, when initial lactose was depleted, a fed-batch phase was initiated. During this phase, a 250 g L^-1^carbon source solution was injected at a 4 mL h^-1 ^rate. The feeding solution composed of either 60% lactose and 40% xylose (W/V) or only lactose. Samples were collected periodically to determine the biomass, carbon and protein concentrations. For both strains, the initial lactose was depleted after 30 h of cultivation. At this stage of the culture, the biomass dry weight concentrations were between 15 to 18 g L^-1 ^and remained steady during the whole fed-batch phase for all cultures whatever conditions were tested. No carbon source accumulation was observed during the whole fed-batch phase.

CL847 strain cultivations were performed in triplicate for the lactose as the only carbon source condition, and in duplicate for the mixed lactose-xylose condition. Only one production was carried out for the Rut-C30 strain.

### Analytical methods

Lactose was assayed by high-performance liquid chromatography on a 7.8 × 300 mm^2 ^HPX-87P column (Biorad) maintained at 85°C, using a Varian Prostar Model 350 HPLC equipped with a refractive index detector. Eluant was helium-degassed distilled water at a flow rate of 0.4 mL min^-1^. Quantification was performed using a solution of 1 g L^-1 ^of lactose as external standard.

Biomass concentration was assayed using a gravimetric method. A culture volume is filtered with a vacuum pump on a dried and preweighed GF/C glass fiber membrane (Wathman). After washing with distilled water, membranes are dried for 48 h at 105°C and weighed.

### Protein extract preparation

Samples were collected around 160 h after start of cultivation. At this stage, protein concentration for all cultures was around 30 g L^-1^. The culture supernatants were harvested by centrifugation for 15 min at 10,800 *g *and 4°C. The supernatants were further clarified on a glass fiber filter GF/F (Whatman, Maidstone, UK) and concentrated and diafiltered against 10 times their volumes of Milli-Q water using a 5 kDa membrane (Amicon system, Millipore Bradford, USA) to eliminate salts. Total protein concentrations were determined in duplicates using the Bio-Rad Dc protein assay kit (Bio-Rad). Aliquots of extracellular protein samples were stored at -80°C for 2DE gel experiments. The same amount of proteins (200 μg) was used for each 2D gel, regardless for the initial supernatant concentration.

### Protein separation by 2D gel electrophoresis

Immobiline DryStrips (18 cm, pH 4–7, Amersham Biosciences) were rehydrated overnight at room temperature with 200 μg of proteins diluted in rehydration solution (DeStreak solution, Amersham Biosciences) supplemented with 2% (v/v) 4–7 IPG buffer and 2.8 mg mL^-1 ^dithiothreitol to a final volume of 350 μL. Isoelectric focusing was performed on a Multiphor II system at 20°C with a 3-phase gradient program: 500 V for 1 Vh, 3500 V for 3 kVh and 3500 V for 27 kVh. Following isoelectric focusing, each strip was equilibrated for 10 min in 10 mL of SDS equilibration buffer (50 mM Tris-HCl pH 6.8, 6 M urea, 30% (v/v) glycerol, 1% (w/v) SDS, a trace of bromophenol blue) containing 25 mM dithiothreitol. A second equilibration step was then performed in the same SDS equilibration buffer containing 250 mM iodoacetamide instead of DTT. The strips were then loaded onto 12% homogeneous acrylamide gels and sealed with 0.5% (w/v) agarose in SDS running buffer (25 mM Tris base, 192 mM glycine, 0.1% (w/v) SDS). The second dimensional separation was performed using an Ettan™ DALT system (Amersham) at 0.5 W/gel and 16°C overnight, followed by 17 W/gel for 3 h. After electrophoresis, the acrylamide gels were either silver-stained for spot picking experiments or stained with Biosafe Coomassie Stain (Biorad) for comparative analysis experiments.

### Protein identification

#### MALDI-TOF mass spectrometry

For protein identification, protein spots were picked up from the gel and silver-stained spots were washed with sodium thiosulfate/potassium ferricyanide, as previously described [22]. All spots were washed, digested by trypsin, extracted and dried as previously described [23]. Spectra were acquired on a MALDI-TOF mass spectrometry Voyager DE-RP (ABI) in positive reflectron mode. Peak list was generated by DataExplorer and manually checked. Identifications were performed using GPMAW software (Lighthouse data).

#### Liquid chromatography tandem mass spectrometry

Spots of protein were excised from a 2D gel and subjected to in-gel tryptic digestion as above. The resulting peptides were extracted and subjected to nanoscale reverse-phase liquid chromatography on a modular LC Packings Ultimate HPLC system equipped with a Famos autosampler and a Switchos microcolumn switching device (LC Packings – a Dionex company, Amsterdam, The Netherlands). The tryptic digest samples were diluted in an aqueous solution containing 0.1% trifluoroacetic acid and pre-concentrated and de-salted at a flow rate of 20 μL min^-1 ^on a 5 mm × 300 μm PepMap C18 precolumn (100 Å, 5 μm, LC Packings). The mobile phase flow from pump C was used to load and wash the sample for 5 min with an aqueous solution containing 0.1% trifluoroacetic acid and 2% acetonitrile. The peptides were then eluted onto a 150 mm × 75 μm analytical PepMap C18 column (100 Å, 3 μm, LC Packings). Chromatographic separation used gradient elution of 95% solution A (acetonitrile/water 2:98, v/v) to 50% solution B (acetonitrile/water 95:5, v/v), both containing 0.08% formic acid and 0.01% trifluoroacetic acid, over 40 min at a flow rate of 200 nL min^-1^. The nanoscale LC eluant from the analytical column was directed to the nanoelectrospray ionization source of a QSTAR^®^XL global hybrid quadrupole/time-of-flight mass spectrometer (Applied Biosystems) run in positive ion mode. A voltage of approximately 2 kV was applied to the spray needle (Picotip Emitter, 360/10 μm, New Objective, MA, USA). Mass spectra were acquired with the Analyst 1.1 software using MS survey for 1 s followed by MS/MS for 3 s. The instrument was calibrated with a multi-point calibration using selected fragment ions that resulted from the collision-induced decomposition (CID) of the C-terminal peptide of β-CN casein 193–209. Data-directed analysis was employed to perform MS/MS analysis on doubly and triply charged precursor ions. Product (fragmentation) ion MS/MS spectra were collected from *m/z *60 to *m/z *2000. Raw data were automatically analyzed on a local server hosting Mascot V.2.1.03.

The *T. reesei *genome database  was used to identify proteins from MS/MS data.

### Image analysis

For comparative studies, each culture sample was independently prepared and used in 2DE in triplicates. To allow an unbiased comparative analysis, Coomassie Blue staining was used instead of silver staining (Biosafe Coomassie Stain, Biorad). The amount of proteins used for 2DE (200 μg) and Coomassie Blue staining was the best compromise between spot-detection sensibility and coloration saturation (data not shown).

Each sample was analyzed in triplicate. Gels were scanned on a calibrated GS800 scanner (Biorad). Images were analyzed using ImageMaster II software (GE Healthcare) using the following workflow. After automatic spot detection, artifacts such as dust or cracks on gels were manually eliminated, and then the weaker spots (individually < 0.05% of the whole gel volume) were eliminated. Remaining spots were then automatically linked to reference spots on a synthetic reference gel to allow comparison between samples.

### Enzymatic assays

All samples were analyzed in duplicate and mean values were calculated. Overall cellulase activity of the samples was measured as Filter Paper (FP) activities using the IUPAC-recommended procedure [24]. Endoglucanase activity was assayed as CMCase activity with CMC (Aqualon) as substrate in 50 mM acetate buffer (pH 4.8) for 30 min at 50°C. Xylanase activity was measured with Oat Spelt Xylan (Sigma) as substrate in the same conditions. For all three activities, sugar release was assayed via the dinitrosalicylic acid method using glucose or xylose as the standard. β-glucosidase activities were determined using 4-nitrophenyl-β-D-glucopyranoside with paranitrophenol as the standard [25].

## Results and discussion

### 2D mapping of T. Reesei CL847 secretome

Cellulase and hemicellulase production is dependent on fungus cultivation conditions [26]. It has been demonstrated that the production of the main cellulases of *Trichoderma *is transcriptionally regulated and carbon source-dependent [8,13]. In order to obtain the fullest complement of the hemicellulolytic enzymatic system, *T. reesei *was grown under conditions promoting the production of both cellulases and hemicellulases. Thus, *T. reesei *was cultivated on a lactose-xylose medium in fed-batch fermentation, since this medium is known to induce the production of both cellulases and hemicellulases in *T. reesei *[27,28].

**Figure 1:**
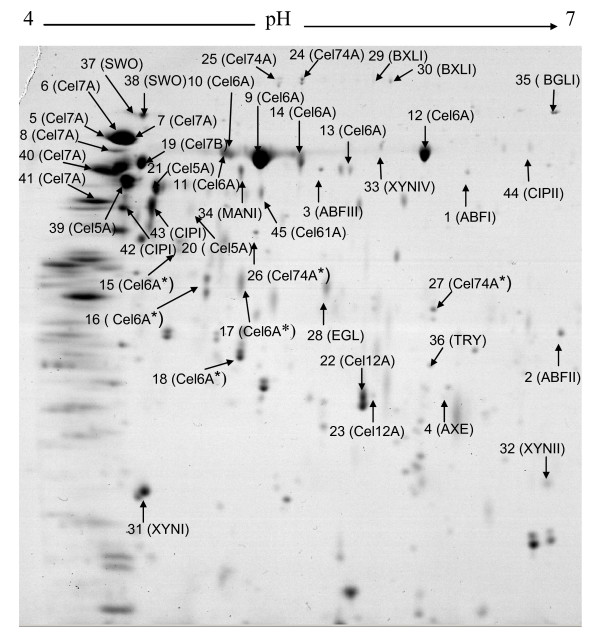
**Coomassie blue-stained 2DE gel of secreted proteins from *T. reesei *CL847 cultivated on xylose–lactose medium**. The protein spots identified are labeled by the protein abbreviations given in Tables 1 and 2. Spots names with asterisks refer to degraded form of proteins.

Total extracellular proteins from the culture supernatant were separated by 2DE. Preliminary investigations using pH 3 to 10 IPG strips revealed that most proteins had pIs < 7. Thus, IPG strips ranging from pH 4 to 7 were chosen for detailed expression analyses to improve the resolution of the proteins spots and facilitate further quantification of individual protein species. The resulting protein maps are shown in Figure 1. Ninety-five distinct protein spots were detected on the 2D gel after staining. The distribution of the protein spots showed that most strongly secreted proteins had an isoelectric point below 6 and a molecular weight above 43 kDa.

Among the 95 protein spots, 36 were identified by MALDI-TOF mass spectrometry (Table 1). To increase the amount of identified proteins, 18 additional spots were analyzed by nanoLC-MS-MS, resulting in the identification of nine further proteins (Table 2). Absence of reliable identification of the remaining protein spots is due to small amounts of biological material and/or post-translational modifications known to affect identification [18]. In most cases, molecular masses observed on 2D gels were higher than the expected masses calculated from the protein sequences, probably because of glycosylation. Several protein spots were assigned to the same protein, suggesting the presence of numerous isoforms and/or degraded forms (Tables 1 and 2).

**Table 1:** Identification of the protein-spots by MALDI-TOF mass spectrometry.

Spot number	Locus^a^	Protein name	Pred^b ^MW/pI	Expt^c ^MW/pI	GPMAW Optimised Score	% sequence coverage
1	ORF_123283	Arabinofuranosidase (ABFI)	51.1/6.0	53/6.3	432	38
2	ORF_76210	Arabinofuranosidase (ABFII)	34.8/6.4	33/6.7	176	34
3	ORF_55319	Arabinofuranosidase (ABFIII)	53.1/5.7	55/5.5	168	16
4	ORF_54219	Candidate acetyl xylan esterase (AXE)	21.9/6.2	27/6.2	222	19
5	ORF_123989	Cellobiohydrolase I (Cel7A)	54.1/4.6	63/4.5	80	7
6	ORF_123989	Cellobiohydrolase I (Cel7A)	54.1/4.6	63/4.4	64	8,8
7	ORF_123989	Cellobiohydrolase I (Cel7A)	54.1/4.6	63/4.6	86	9
8	ORF_123989	Cellobiohydrolase I (Cel7A)	54.1/4.6	57/4.7	107	13
9	ORF_72567	Cellobiohydrolase II (Cel6A)	49.6/5.1	56/5.2	210	24
10	ORF_72567	Cellobiohydrolase II (Cel6A)	49.6/5.1	58/5.0	153	11
11	ORF_72567	Cellobiohydrolase II (Cel6A)	49.6/5.1	59/4.8	207	18
12	ORF_72567	Cellobiohydrolase II (Cel6A)	49.6/5.1	58/6.0	165	17
13	ORF_72567	Cellobiohydrolase II (Cel6A)	49.6/5.1	55/5.6	125	11
14	ORF_72567	Cellobiohydrolase II (Cel6A)	49.6/5.1	55/5.4	77	4
15	ORF_72567	Cellobiohydrolase II (Cel6A)	49.6/5.1	42/4.7	157	20
16	ORF_72567	Cellobiohydrolase II (Cel6A)	49.6/5.1	38/4.9	279	17
17	ORF_72567	Cellobiohydrolase II (Cel6A)	49.6/5.1	38/5.0	307	20
18	ORF_72567	Cellobiohydrolase II (Cel6A)	49.6/5.1	30/5.1	279	17
19	ORF_122081	Endoglucanase I (Cel7B)	48.2/4.7	55/4.6	57	9
20	ORF_120312	Endoglucanase II (Cel5A)	44.1/5.0	43/4.8	160	31
21	ORF_120312	Endoglucanase II (Cel5A)	44.1/5.0	48/4.6	64	12
22	ORF_123232	Endoglucanase III (Cel12A)	25.1/6.7	25/5.7	185	19
23	ORF_123232	Endoglucanase III (Cel12A)	25.1/6.7	26/57	185	19
24	ORF_49081	Xyloglucanase (Cel74A)	87.1/5.4	96/5.4	201	16
25	ORF_49081	Xyloglucanase (Cel74A)	36.2/8.7	96/5.3	520	26
26	ORF_49081	Xyloglucanase (Cel74A)	36.2/8.7	43/5.2	406	21
27	ORF_49081	Xyloglucanase (Cel74a)	36.2/8.7	35/6.0	354	12
28	ORF_27554	Candidate Endoglucanase (EGL)	36.2/8.7	36/5.5	87	15
29	ORF_121127	Xylosidase I (BXLI)	87.2/5.5	97/5.6	208	12
30	ORF_121127	Xylosidase I (BXLI)	87.2/5.5	97/5.7	203	15
31	ORF_74223	Xylanase I (XYNI)	24.6/5.0	21/4.6	85	16
32	ORF_123818	Xylanase II (XYNII)	24.1/7.9	21/6.6	212	27
33	ORF_111849	Xylanase IV (XYNIV)	52.8/5.7	55/5.6	125	13
34	ORF_56996	Mannanase I (MANI)	40.2/5.1	53/5.1	147	17
35	ORF_76672	β-Glucosidase (BGLI)	78.4/6.4	81/6.7	440	32
36	ORF_73897	Trypsin-like protease (TRY)	26.4/5.8	29/6.0	92	28

^a ^From

^b^Predicted MW (kDa) and pI according to the sequence

^C^Experimental MW (kDa) and pI

**Table 2:** Identification of the protein-spots by Nano-LC MSMS mass spectrometry.

Spot number	Locus^a^	Protein name	Pred^b ^MW/pI	Expt^c ^MW/pI	Number of peptides matched	Global Score	% sequence coverage
37	ORF_123992	Swollenin (SWO)	51.5/4.8	80/4.6	8	296	20.3
38	ORF_123992	Swollenin (SWO)	51.5/4.8	80/4.7	6	252	20.3
19	ORF_1220081	Endoglucanase I (Cel7B)	48.2/4.7	55/4.6	13	477	31.6
39	ORF_120312	Endoglucanase II (Cel5A)	44.1/5.0	50/4.5	9	181	32.3
21	ORF_120312	Endoglucanase II (Cel5A)	44.1/5.0	48/4.6	13	451	39.5
40	ORF_123989	Cellobiohydrolase I (Cel7A)	54.1/4.6	55/4.4	4	114	10.1
41	ORF_123989	Cellobiohydrolase I (Cel7A)	54.1/4.6	47/4.4	2	65	3.1
42	ORF_73638	Cellulose binding protein (CIPI)	32.9/4.9	43/4.6	5	171	11.1
43	ORF_73638	Cellulose binding protein (CIPI)	32.9/4.9	47/4.5	9	250	39.9
44	ORF_123940	Cellulose binding protein (CIPII)	48.3/7.0	57/6.6	7	281	15.2
45	ORF_73643	Endoglucanase IV (Cel61A)	35.5/5.3	47/5.2	1	36^d^	3.8

^a ^From

^b^Predicted MW (kDa) and pI according to the sequence

^C^Experimental MW (kDa) and pI

^d^Individual peptide scores > 26 indicate identity or extensive homology (*p *> 0.05)

As expected, most of the identified proteins were related to biomass degradation and were assigned to cellulases and hemicellulases. Cellobiohydrolases Cel7A and Cel6A were the two most abundantly secreted proteins. These proteins are known to account for 70 to 80% of the total *T. reesei *cellulases [4,29], consistent with the high intensity of the corresponding protein spots observed on the gel. The only β-glucosidase identified on the gel was BGLI, in accordance with reports of the other β-glucosidases being either intra-cellular, membrane-anchored, or playing only a minor role in cellulose hydrolysis [6]. Four out of the five known endoglucanases were also identified, but one of them, endoglucanase Cel61A, was only identified with a single peptide and thus should be considered provisional (Table 2). The minor endoglucanase Cel45A of *T. reesei *[30] was not identified, probably because of its highly acidic pI. This secretome analysis also revealed the expression of the ORF_27554 product annotated as candidate endoglucanase in the *T. reesei *genome database. The similarity between the observed and predicted molecular weight for this new endoglucanase (Table 1) suggests that this protein is only sparsely glycosylated. In addition, the product of the gene *cel74a *[6] was detected on the protein map. This enzyme, formerly endoglucanase VI, has been characterized as a xyloglucanase. The observation that several spots matched to this protein supports previous data that there are multiple isoforms of this enzyme [31].

We also identified some major components of the hemicellulolytic system of *T. reesei*: β-xylosidases, xylanases and arabinofuranosidase (Table 1). Three out of the four known xylanases were identified. The last xylanase, XYNIII, focalizes at a pH around 8 and is outside the range of our pH 4 to 7 gels (data not shown). Furthermore, we did not identify any galactosidases, which is surprising given that these proteins are purported to be induced by lactose [32]. It is not unlikely that this protein corresponded to one of the minor unidentified spots. The present study highlights the production of a putative arabinofuranosidase (ORF_55319, ABFIII Table 1). As stated previously for the putative endoglucanase, the close correlation between the observed and predicted molecular weight of this putative arabinofuranosidase suggests that this enzyme is also sparsely glycosylated. *T. reesei *is thus able to produce at least three different arabinofuranosidases. Two of them have already been described in a purification study (ABFI) [33] and in cDNA analysis (ABFII) [13]. This work provides evidence for the production of both ABFII and a novel third α-L-arabinofuranosidase (ORF_55319, ABFIII) not reported previously. Apart from cellulases and hemicellulases, non-hydrolytic proteins including CIPI, CIPII and swollenin were identified (Table 2), providing new evidence for the production and secretion of these proteins. CIPI and CIPII were discovered during the *T. reesei *genome sequencing program as proteins with a cellulose-binding domain, but no other functional domain, such as a glycosyl hydrolase domain, could be found [13]. However, a recent phylogenetic analysis suggests they share close relationships with cellulases, which adds support to the potential roles of these genes in biomass degradation [14]. Proteolytic enzymes such as trypsin were also found at low levels (0.2%). This may explain the presence of some altered proteins, especially Cel6A, whose molecular weight in the gels was lower than expected (Figure 1). Heterogeneity of cellobiohydrolases on PAGE-SDS has already been reported, and explained by glycosylation and proteolysis [19].

In total, 22 biomass-degrading enzymes were identified on our gels, to be compared with the previous study of Vinzant et al (2001) [19] where only 10 enzymes were identified. An analysis of the lactose-xylose 2D gel image by ImageMaster II software indicated that the identified proteins account for 83% of all visible proteins in the gel in terms of spot volume. This percentage rises to 93% for the secretome of *T. reesei *CL847 grown on lactose alone.

### Comparison between secretomes of CL847 and Rut-C30 produced on lactose

Rut-C30 has for decades been the reference cellulase-overproducing strain in academic publications. This strain, like CL847, has been obtained through random mutagenesis and subsequent screening. The last common ancestor of these two strains is the reference *T. reesei *strain QM6a. CL847 was further evolved from strain QM9414. Enzymatic activities vary significantly between these two strains (Table 3). In the same culture conditions, Rut-C30 has a slightly higher FPase and CMCase-specific activity, while xylanase and β-glucosidase activities are significantly higher for CL847 strain (respectively ×2.7 and ×1.5). Two mutations were identified in Rut-C30. Firstly, a mutation in *cre1*, a gene encoding a transcription factor mediating glucose repression for cellulase production was discovered. This frameshift mutation leads to a truncated protein that might account for some increase in cellulase production in this strain [34]. Secondly, a frameshift mutation was observed for glucosidase II alpha subunit, leading to defective extracellular protein glycosylation [35]. However, it is almost certain that these are not the only mutations affecting this strain. As Rut-C30 grown on lactose and xylose failed to produce cellulases, secretomes of Rut-C30 and CL847 were produced with lactose as the only carbon source.

**Table 3:** Routinely obtained specific activities for cellulase preparations used in present work.

	FPase μmol glucose/min/mg prot	CMCase μmol glucose/min/mg prot	Xylanase μmol xylose/min/mg prot	β-glucosidase μmol PNP/min/mg prot
CL847 Lact-Xyl	0.36 (± 0.02)	0.53 (± 0.06)	55 (± 5.5)	1121 (± 6)

CL847 Lact	0.39 (± 0.025)	0.46 (± 0.01)	34 (± 1.5)	13919 (± 373)

Rut-C30 Lact	0.43 (± 0.009)	0.58 (± 0.05)	12.5 (± 0.8)	9019 (± 96)

Numbers in brackets are standard deviations obtained for the same preparation with duplicates. 'Lact' stands for production induced with Lactose alone while 'Lact-Xyl' stands for production induced with mixed lactose and xylose (see Methods section). FPase stands for Filter Paper activity and CMCase stands for activity on Carboxy-methyl-cellulose.

As for proteome map construction, samples were taken during the late fed-batch production phase (around 160 h) for each sample. At this stage of the production, protein concentrations were around 30 g L^-1 ^for both strains.

**Figure 2:**
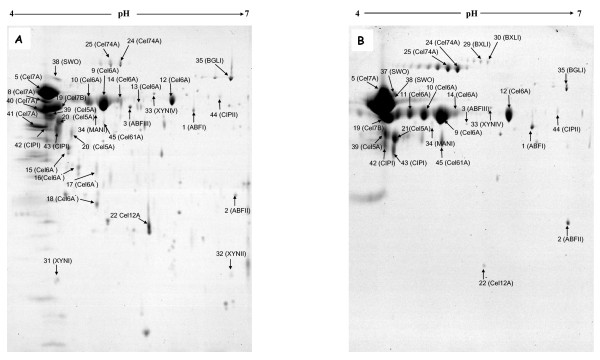
**Coomassie blue-stained 2DE gel of secreted proteins from *T. reesei *CL847 (A) and Rut-C30 (B) cultivated on lactose medium**. The protein spots identified are labeled by the protein abbreviations given in Tables 1 and 2.

**Figure 3:**
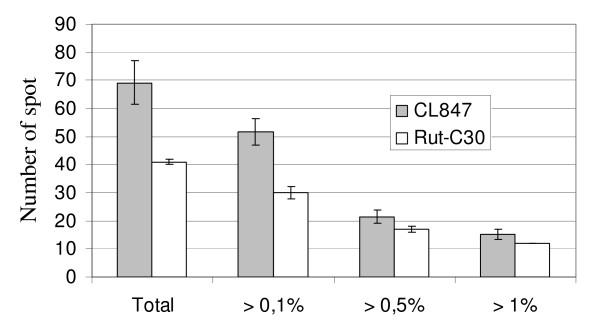
**Spot volume distribution of Rut-C30 and CL847 secretomes**. Standard deviations are calculated from three replicates. Differences between the two strains can be related to both small spots, which are more abundant in CL847, and isoforms, equally more abundant in this strain.

**Figure 4:**
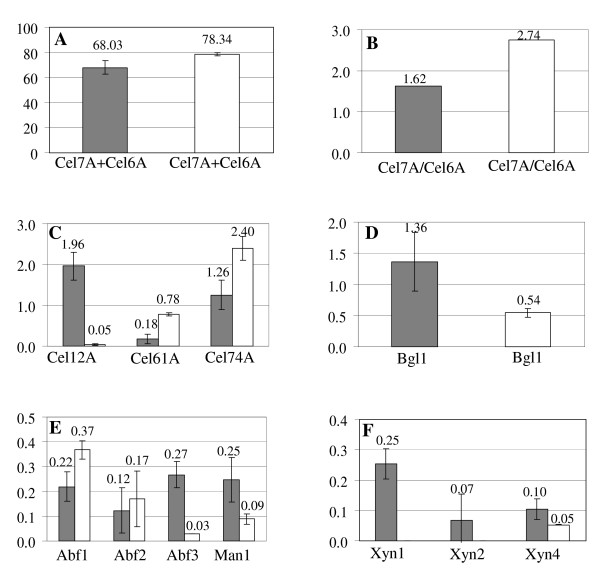
**Comparative Analysis of Rut-C30 (white histograms) and CL847 (grey histograms) secretomes**. Values are expressed in %Volume except for B where it is a %Cel7A/%Cel7B ratio. **A: **Volume of total cellobiohudrolases; **B: **Cel7A-to-Cel6A ratio; **C: **Secondary endoglucanases; **D: **β-glucosidase; **E: **Non-xylolytic hemicellulases and **F: **Xylanases.

The 2DE profiles of CL847 and Rut-C30 grown on lactose were very different, in terms of both spot numbers and protein composition (Figure 2). CL847 2DE reveals many more protein spots that Rut-C30, especially in minor spots corresponding to less than 0.5% of total spots volume (Figure 3), and consequently most of these spots are unidentified or correspond to degradation forms absent in Rut-C30. Differences in protein spots representing a higher percentage of the total spot volume are due to the presence of several Cel7A isoforms for CL847, while a single and bigger spot is visible for Rut-C30. In contrast, Cel6A isoform profiles were similar. Protein spot quantitation revealed that Rut-C30 has 10% more total cellobiohydrolases than CL847 (Figure 4A). This is related to a higher Cel7A level in this strain (57.4% in Rut-C30 versus 42.1% in CL847), since Cel6A levels were not significantly different between the two strains. As a consequence, the Cel7A-to-Cel6A ratio is much higher in Rut-C30 than in CL847 (Figure 4B). This is in disagreement with the widespread hypothesis that Cel7A and Cel6A are co-regulated [16,36]. Nevertheless, we cannot rule out the possibility that this change in ratio could be due to a higher level of degradation of Cel7A in CL847. In contrast with cellobiohydrolases levels, the relative amount of BGLI produced by CL847 is twice as much as compared to Rut-C30 (Figure 4D), which is reflected in β-glucosidases activities for these strains in similar conditions (Table 3). No significant differences were observed for endoglucanases Cel7B and Cel5A. However, this area of the gels is heavily crowded, especially for CL847, and any quantification must be taken with caution (Figure 2). Contrast was more pronounced for minor endoglucanases. CL847 produces around 2% Cel12A, while it is almost undetectable in Rut-C30 samples. Cel61A and Cel74A levels were much higher in Rut-C30 (Figure 4C). These results contrast with those of Foreman et al (2003) [13], where endoglucanase co-expression was observed at the mRNA level, prompting the authors to propose co-regulation of these enzymes. We observed no such events at protein level, but the differences may be due to the different strains and culture conditions used in our work and Foreman's. Another point to highlight is that the EGL and AXEIII are also absent in the both strains cultivated on lactose. The β-xylosidase, BXLI, is only present in Rut-C30, although at a very low percentage (0.2%). This is consistent with reports of a low constitutive expression of the BXLI protein in Rut-C30 [10]. The only xylanase expressed in Rut-C30 was XYNIV, secreted at a similar level to CL847 (Figure 4F). In contrast, in CL847, XYNIV only figured as one of the minor xylanases. This suggests that expression of xylanases XYNI, XYNII and XYNIV is different in the two studied *Trichoderma *strains and that these different expression pathways were not equally affected by the mutations that led to the CL847 and Rut-C30 phenotypes. Globally, Rut-C30 has a lower xylan-related enzyme secretion while CL847 secretes a more diversified set of enzymes. Other hemicellulase levels also showed marked differences. While ABFII levels were comparable and ABFIII level was tenfold higher in CL847, ABFI was slightly over-produced in Rut-C30 (Figure 4E). As for xylanases, the results suggest that the role of these proteins is not equivalent and that they are not co-regulated. The only mannanase of *T. reesei *was expressed twice more in CL847 than in Rut-C30 (Figure 4E). Among non-cellulolytic enzymes, except for the absence of CIPII, there was no significant detectable difference in CIPI and SWO. Finally, trypsin was absent in Rut-C30. The presence of proteases may explain the observation of degraded forms of proteins in CL847 and the much higher number of spots, especially in the low molecular weight region of the gels.

These results fit nicely with data obtained from specific enzymatic activities (Table 3): the lower FPase activity in CL847 can be related to the lower amount of cellobiohydrolases in this strain. While it is difficult to link the CMCase activity differences to any specific protein, since many enzymes exhibit endoglucanase activity, the higher β-glucosidase and xylanase specific activities are consistent with the higher BGLI and xylanases activities in the CL847 cellulase productions (Figure 4).

## Conclusion

A total of 22 extracellular protein species of *T. reesei *was identified. Most of the corresponding proteins were involved in lignocellulose degradation. In addition to the reference map of the secreted proteins, we describe hitherto unreported experimental evidence of the expression of a new putative endoglucanase and a new putative arabinofuranosidase.

The last common ancestor of CL847 and Rut-C30 is the originally isolated strain QM6a. This secretome study shows that mutagenesis, in addition to improving the secretion capacities of cellulases, also seems to lead to different enzyme cocktail compositions. The more diversified secretome of CL847 suggests that this strain may be a more general hypersecretory strain while Rut-C30 may be more cellulase-oriented. It suggests that Rut-C30 and CL847 were obtained using a totally different 'mutation route' towards becoming efficient producers. On the genetic regulation level, the only known transcription factors are the XYRI and ACEII activators and the CREI and ACEI repressors [8], all of which act at global level. Contrasts in secretome profiles, such as those observed in CL847 and Rut-C30 in this research, could potentially be attributed to chromosomal rearrangements or chromatin structure [35], making it particularly tricky to identify the mutations involved without extensive genomic investigation. Moreover, studying secretomes produced in different conditions for the same strains will provide greater insight into secretion behavior and cellulase regulation.

## Competing interests

The authors declare that they have no competing interests.

## Authors' contributions

IHG and AM carried out the 2D gel electrophoresis experiments and drafted the manuscript. AD analyzed the MALDI-TOF mass spectra using the GPMAW software and helped with manuscript preparation. GJ participated in the protein identification by liquid chromatography-tandem mass spectrometry and helped with manuscript preparation. DM carried out the protein identification by liquid chromatography-tandem mass spectrometry. SL performed the MALDI-TOF mass spectrometry measurements. HM performed 2D gels, image analysis and spot picking for protein identification. JCS reviewed and commented on the manuscript. FM and MA directed the overall study and drafting of the manuscript. All authors read and approved the final manuscript.
